# 2-[(Eth­oxy­carbonothio­yl)sulfan­yl]acetic acid

**DOI:** 10.1107/S1600536811017703

**Published:** 2011-05-20

**Authors:** Shude Xiao, Renpeng Gu, Paul A. Charpentier

**Affiliations:** a Department of Chemical and Biochemical Engineering, Faculty of Engineering, The University of Western Ontario, London, Ontario, Canada N6A 5B9

## Abstract

In the title compound, C_5_H_8_O_3_S_2_, the C—S and C—O bonds in the xanthate unit are shorter than those linked to it. In the crystal, inversion dimers linked by pairs of O—H⋯O hydrogen bonds occur.

## Related literature

For general background to the synthesis and applications of the title compound, see: Stenzel *et al.* (2003 ▶); Moad *et al.* (2005 ▶, 2008 ▶). For its applications in polymerization, see: Coote & Radom (2004 ▶); Simms *et al.* (2005 ▶); Russum *et al.* (2005 ▶); Assem *et al.* (2007 ▶); Wang *et al.* (2010 ▶). For similar structures, see: Xiao & Charpentier (2010 ▶, 2011 ▶).
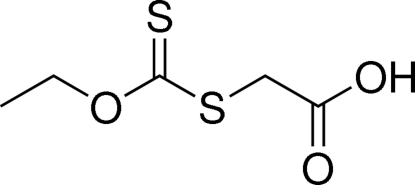


## Experimental

### 

#### Crystal data

C_5_H_8_O_3_S_2_
*M* *_r_* = 180.23
Monoclinic, 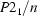

*a* = 4.7387 (2) Å
*b* = 14.7836 (8) Å
*c* = 11.9013 (6) Å
β = 100.845 (3)°
*V* = 818.86 (7) Å^3^
*Z* = 4
Mo *K*α radiation
μ = 0.60 mm^−1^
*T* = 150 K
0.08 × 0.03 × 0.03 mm

#### Data collection

Bruker APEXII CCD diffractometer
Absorption correction: multi-scan (Blessing, 1995 ▶) *T*_min_ = 0.952, *T*_max_ = 0.982
39762 measured reflections
3582 independent reflections
2343 reflections with *I* > 2σ(*I*)
*R*_int_ = 0.092

#### Refinement

*R*[*F* ^2^ > 2σ(*F* ^2^)] = 0.045
*wR*(*F* ^2^) = 0.111
*S* = 1.01
3582 reflections
93 parameters
H-atom parameters constrained
Δρ_max_ = 0.31 e Å^−3^
Δρ_min_ = −0.37 e Å^−3^

### 

Data collection: *APEX2* (Bruker, 2008 ▶); cell refinement: *SAINT* (Bruker, 2008 ▶); data reduction: *SAINT*; program(s) used to solve structure: *SHELXS97* (Sheldrick, 2008 ▶); program(s) used to refine structure: *SHELXL97* (Sheldrick, 2008 ▶); molecular graphics: *SHELXTL* (Sheldrick, 2008 ▶); software used to prepare material for publication: *SHELXTL*.

## Supplementary Material

Crystal structure: contains datablocks global, I. DOI: 10.1107/S1600536811017703/ng5142sup1.cif

Structure factors: contains datablocks I. DOI: 10.1107/S1600536811017703/ng5142Isup2.hkl

Supplementary material file. DOI: 10.1107/S1600536811017703/ng5142Isup3.cml

Additional supplementary materials:  crystallographic information; 3D view; checkCIF report

## Figures and Tables

**Table 1:** Hydrogen-bond geometry (Å, °)

*D*—H⋯*A*	*D*—H	H⋯*A*	*D*⋯*A*	*D*—H⋯*A*
O2—H2⋯O3^i^	0.84	1.81	2.645 (2)	175

Symmetry code: (i) 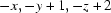.

**Fig. 1.**
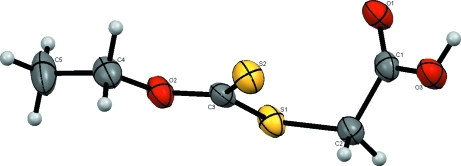
View of the title compound (50% probability displacement ellipsoids).

**Fig. 2.**
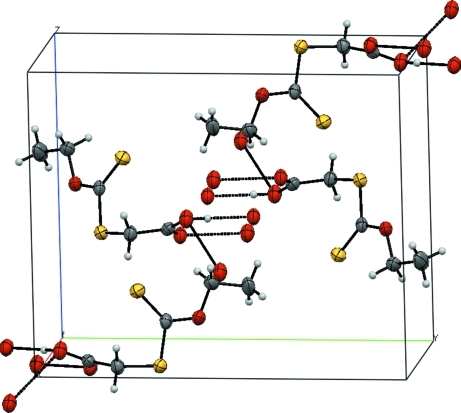
Packing diagram of the structure with H-bonds.

## supplementary crystallographic information

### Comment

Reversible-deactivation radical polymerization (RDRP) of vinyl acetate (VAc) has been a challenge. Methyl 2-(ethoxycarbonothioylthio)acetate was investigated for reversible addition-fragmentation chain transfer (RAFT) polymerization of VAc (Moad *et al.*, 2005, 2008; Stenzel *et al.*, 2003; Coote & Radom, 2004), and was successfully applied in emulsion polymerizations (Simms *et al.*, 2005; Russum *et al.*, 2005). Thanks to the similarity of the molecular structures, 2-(ethoxycarbonothioylthio)acetic acid not only provides a carboxylic acid functionality but also works as the RAFT-CTA for VAc in RDRP. This RAFT-CTA has also found applications in the RAFT polymerization of other monomers (Assem *et al.*, 2007; Wang *et al.*, 2010). Compounds of similar structures were reported previously (Xiao & Charpentier, 2010, 2011).

### Experimental

Potassium *O*-ethyl dithiocarbonate 13.6 g was dissolved in THF 50 ml, and then mixed with 2-bromoacetic acid 6.9 g / THF 20 ml. The reaction was carried out at room temperature for 2 days. Excess hexanes was applied to the mixture and the precipitates were filtered off, followed by evaporating the solvents using a rotary evaporator. The light yellow oil was further purified by extraction and recrystallization with hexanes, and colorless crystals were obtained. m.p.: 56.4 °C(DSC). MS: 179.9917.

### Refinement

The structure was solved and refined using the Bruker *SHELXTL* Software Package, using the space group P 1 21/n 1, with *Z* = 4 for the formula unit, C_5_H_8_O_3_S_2_. All of the non-hydrogen atoms were refined with anisotropic thermal parameters. The hydrogen atom positions were calculated geometrically and were included as riding on their respective carbon atoms. The final anisotropic full-matrix least-squares refinement on F^2^ with 93 variables converged at R1 = 4.54%, for the observed data and wR2 = 11.08% for all data. The goodness-of-fit was 1.005. The largest peak in the final difference electron density synthesis was 0.309 e^-^/Å^3^ and the largest hole was -0.368 e^-^/Å^3^ with an RMS deviation of 0.077 e^-^/Å^3^. On the basis of the final model, the calculated density was 1.462 g/cm^3^ and F(000), 376 e^-^.

### Crystal data

**Table:**

C_5_H_8_O_3_S_2_	*F*(000) = 376
*M**_r_* = 180.23	*D*_x_ = 1.462 Mg m^−^^3^
Monoclinic, *P*2_1_/*n*	Mo *K*α radiation, λ = 0.71073 Å
Hall symbol: -P 2yn	Cell parameters from 4468 reflections
*a* = 4.7387 (2) Å	θ = 2.2–25.6°
*b* = 14.7836 (8) Å	µ = 0.60 mm^−^^1^
*c* = 11.9013 (6) Å	*T* = 150 K
β = 100.845 (3)°	Cube, colourless
*V* = 818.86 (7) Å^3^	0.08 × 0.03 × 0.03 mm
*Z* = 4	

### Data collection

**Table:**

Bruker APEXII CCD diffractometer	3582 independent reflections
Radiation source: fine-focus sealed tube	2343 reflections with *I* > 2σ(*I*)
graphite	*R*_int_ = 0.092
φ and ω scans	θ_max_ = 35.0°, θ_min_ = 2.2°
Absorption correction: multi-scan (Blessing, 1995)	*h* = −7→7
*T*_min_ = 0.952, *T*_max_ = 0.982	*k* = −23→23
39762 measured reflections	*l* = −19→18

### Refinement

**Table:**

Refinement on *F*^2^	Primary atom site location: structure-invariant direct methods
Least-squares matrix: full	Secondary atom site location: difference Fourier map
*R*[*F*^2^ > 2σ(*F*^2^)] = 0.045	Hydrogen site location: inferred from neighbouring sites
*wR*(*F*^2^) = 0.111	H-atom parameters constrained
*S* = 1.01	*w* = 1/[σ^2^(*F*_o_^2^) + (0.0294*P*)^2^ + 0.6076*P*] where *P* = (*F*_o_^2^ + 2*F*_c_^2^)/3
3582 reflections	(Δ/σ)_max_ = 0.001
93 parameters	Δρ_max_ = 0.31 e Å^−^^3^
0 restraints	Δρ_min_ = −0.37 e Å^−^^3^

### Special details

**Table:**

Geometry. All e.s.d.'s (except the e.s.d. in the dihedral angle between two l.s. planes) are estimated using the full covariance matrix. The cell e.s.d.'s are taken into account individually in the estimation of e.s.d.'s in distances, angles and torsion angles; correlations between e.s.d.'s in cell parameters are only used when they are defined by crystal symmetry. An approximate (isotropic) treatment of cell e.s.d.'s is used for estimating e.s.d.'s involving l.s. planes.
Refinement. Refinement of *F*^2^ against ALL reflections. The weighted *R*-factor *wR* and goodness of fit *S* are based on *F*^2^, conventional *R*-factors *R* are based on *F*, with *F* set to zero for negative *F*^2^. The threshold expression of *F*^2^ > σ(*F*^2^) is used only for calculating *R*-factors(gt) *etc*. and is not relevant to the choice of reflections for refinement. *R*-factors based on *F*^2^ are statistically about twice as large as those based on *F*, and *R*- factors based on ALL data will be even larger.

### Fractional atomic coordinates and isotropic or equivalent isotropic displacement parameters (Å^2^)

**Table:**

	*x*	*y*	*z*	*U*_iso_*/*U*_eq_	
S1	−0.08173 (11)	0.19417 (3)	1.01439 (4)	0.02745 (11)	
S2	−0.03755 (11)	0.25308 (3)	0.77468 (4)	0.03030 (12)	
O1	0.1868 (3)	0.10724 (9)	0.88947 (11)	0.0300 (3)	
O2	−0.3267 (3)	0.44372 (9)	0.93538 (13)	0.0329 (3)	
H2	−0.2443	0.4941	0.9464	0.049*	
O3	0.0854 (3)	0.39432 (9)	1.04203 (12)	0.0306 (3)	
C1	0.4719 (6)	−0.00588 (15)	0.8227 (2)	0.0510 (6)	
H1A	0.6206	0.0051	0.8906	0.077*	
H1B	0.5624	−0.0255	0.7592	0.077*	
H1C	0.3409	−0.0531	0.8396	0.077*	
C2	0.3067 (5)	0.07965 (13)	0.79013 (17)	0.0327 (4)	
H2A	0.4352	0.1273	0.7699	0.039*	
H2B	0.1508	0.0690	0.7234	0.039*	
C3	0.0329 (4)	0.18315 (11)	0.88283 (14)	0.0236 (3)	
C4	−0.3109 (4)	0.29105 (12)	0.98778 (16)	0.0276 (3)	
H4A	−0.4369	0.2837	0.9120	0.033*	
H4B	−0.4359	0.2923	1.0457	0.033*	
C5	−0.1604 (4)	0.38094 (12)	0.98990 (14)	0.0239 (3)	

### Atomic displacement parameters (Å^2^)

**Table:**

	*U*^11^	*U*^22^	*U*^33^	*U*^12^	*U*^13^	*U*^23^
S1	0.0365 (2)	0.02475 (19)	0.02263 (19)	−0.00361 (17)	0.00955 (16)	0.00040 (15)
S2	0.0354 (3)	0.0337 (2)	0.02195 (19)	−0.00021 (19)	0.00584 (17)	0.00340 (16)
O1	0.0375 (7)	0.0262 (6)	0.0284 (6)	0.0002 (5)	0.0118 (5)	0.0004 (5)
O2	0.0272 (7)	0.0267 (6)	0.0423 (8)	0.0001 (5)	0.0000 (6)	−0.0006 (6)
O3	0.0266 (6)	0.0272 (6)	0.0356 (7)	−0.0025 (5)	−0.0001 (5)	0.0014 (5)
C1	0.0734 (18)	0.0294 (10)	0.0603 (15)	0.0068 (11)	0.0385 (14)	−0.0004 (10)
C2	0.0384 (11)	0.0299 (9)	0.0332 (9)	−0.0048 (8)	0.0151 (8)	−0.0068 (7)
C3	0.0242 (8)	0.0242 (7)	0.0228 (7)	−0.0060 (6)	0.0050 (6)	−0.0028 (6)
C4	0.0259 (8)	0.0298 (9)	0.0289 (8)	−0.0038 (7)	0.0098 (7)	−0.0011 (7)
C5	0.0237 (8)	0.0269 (8)	0.0223 (7)	−0.0011 (6)	0.0075 (6)	−0.0020 (6)

### Geometric parameters (Å, °)

**Table:**

S1—C3	1.7588 (17)	C1—H1A	0.9800
S1—C4	1.789 (2)	C1—H1B	0.9800
S2—C3	1.6356 (18)	C1—H1C	0.9800
O1—C3	1.332 (2)	C2—H2A	0.9900
O1—C2	1.463 (2)	C2—H2B	0.9900
O2—C5	1.308 (2)	C4—C5	1.506 (2)
O2—H2	0.8400	C4—H4A	0.9900
O3—C5	1.228 (2)	C4—H4B	0.9900
C1—C2	1.499 (3)		
			
C3—S1—C4	101.27 (9)	H2A—C2—H2B	108.6
C3—O1—C2	118.58 (14)	O1—C3—S2	127.40 (13)
C5—O2—H2	109.5	O1—C3—S1	106.40 (12)
C2—C1—H1A	109.5	S2—C3—S1	126.20 (11)
C2—C1—H1B	109.5	C5—C4—S1	115.70 (13)
H1A—C1—H1B	109.5	C5—C4—H4A	108.4
C2—C1—H1C	109.5	S1—C4—H4A	108.4
H1A—C1—H1C	109.5	C5—C4—H4B	108.4
H1B—C1—H1C	109.5	S1—C4—H4B	108.4
O1—C2—C1	106.83 (17)	H4A—C4—H4B	107.4
O1—C2—H2A	110.4	O3—C5—O2	124.18 (16)
C1—C2—H2A	110.4	O3—C5—C4	123.47 (16)
O1—C2—H2B	110.4	O2—C5—C4	112.25 (15)
C1—C2—H2B	110.4		

### Hydrogen-bond geometry (Å, °)

**Table:**

*D*—H···*A*	*D*—H	H···*A*	*D*···*A*	*D*—H···*A*
O2—H2···O3^i^	0.84	1.81	2.645 (2)	175

Symmetry codes: (i) −*x*, −*y*+1, −*z*+2.
